# Clinical characteristics, management, and outcomes of severe tetanus in the intensive care unit

**DOI:** 10.1186/s12879-026-13697-6

**Published:** 2026-05-28

**Authors:** Bin-bin Gu, Feng-feng Zhu, Di Zou, Jian Ding, Teng Zhou, Lin Yao, Xing-hua Shen

**Affiliations:** 1 Intensive Care Unit, The Fifth People’s Hospital of Suzhou, The Affiliated Infectious Disease Hospital of Soochow University, 10 Guangqian Road, Suzhou, 215000 China; 2 Department of Pulmonary, The Fifth People’s Hospital of Suzhou, The Affiliated Infectious Disease Hospital of Soochow University, 10 Guangqian Road, Suzhou, 215000 China; 3 Soochow University Affiliated Infectious Disease Hospital, 10 Guangqian Road, Suzhou, 215000 China

**Keywords:** Tetanus, Intensive care unit, Mechanical ventilation, Infectious complications

## Abstract

**Background:**

Tetanus is a life-threatening but vaccine-preventable disease. Despite existing preventive measures, severe tetanus continues to impose a significant burden in low- and middle-income countries. However, contemporary data on the clinical characteristics, treatment strategies, complications, and prognosis of patients with severe tetanus remain limited.

**Methods:**

We conducted a retrospective observational cohort study of adult patients with severe tetanus (Ablett grade III or IV) admitted to the intensive care unit (ICU) of a tertiary infectious disease hospital from January 2014 to December 2024. Patient demographics, clinical manifestations, laboratory findings, treatment strategies, complications, and outcomes were extracted from electronic medical records and summarized using descriptive statistical methods.

**Results:**

A total of 36 patients were included, the majority were older men from rural areas, and relatively few had received post-exposure prophylaxis. In the early stage of the disease, dysphagia and generalized muscle hypertonia were more frequent than classic symptoms such as trismus and opisthotonos. Most patients required tracheostomy, deep sedation, neuromuscular blockade, and invasive mechanical ventilation. Infectious complications were common, particularly pulmonary infections (80.6%). More than half of the patients (55.6%) had culture results indicating antimicrobial resistance. The median ICU length of stay was 27.5 days, and the in-hospital mortality rate was 16.7%.

**Conclusion:**

Although severe tetanus is potentially fatal, outcomes can be improved with advanced intensive care management. Early intervention, comprehensive supportive therapy, strict infection control, and strengthened tetanus vaccination and post-exposure prophylaxis strategies are crucial.

**Supplementary Information:**

The online version contains supplementary material available at 10.1186/s12879-026-13697-6.

## Introduction

Tetanus is a vaccine-preventable disease caused by *Clostridium tetani*, characterized by painful muscle spasms, rigidity, and autonomic instability [1]. Although tetanus can be largely prevented with timely vaccination programs and adequate wound management, it remains prevalent in many low- and middle-income countries, particularly among the elderly and unvaccinated [2]. The World Health Organization has estimated that tens of thousands of tetanus-related deaths occurred annually in the early 2000s, with substantial declines in recent years due to expanded immunization programs [3]. Patients with severe tetanus often require admission to the ICU, prolonged mechanical ventilation, deep sedation, and extensive supportive care, placing a heavy burden on the healthcare system [4].

The clinical outcomes of patients with tetanus has improved due to advances in intensive care. Mortality is down from around 40–50% to less than 20%. However, severe tetanus patients impose a significant burden on intensive care resources. This group usually requires prolonged mechanical ventilation, tracheostomy and complex sedation [5]. Hospital-acquired infections, especially ventilator-associated pneumonia, are common complications in severe patients, prolonging ICU stays and increasing morbidity and mortality [6]. Current guidelines recommend aggressive supportive care, but evidence for best management strategies across different clinical situations remains limited [7].

In this study, we did a comprehensive exploration of the clinical features, therapeutic management, complications, and outcomes with severe tetanus. It aimed to enhance the understanding of effective clinical strategies and to provide useful references for improving the prognosis of severe tetanus.

## Methods

### Patients

All patients with severe tetanus were admitted to the intensive care unit (ICU) of The Fifth People’s Hospital of Suzhou. The hospital is the only tertiary infectious disease medical centre in Suzhou, and it serves a population of about 12 million. The intensive care unit has 19 beds and provides care for urban and rural residents of Suzhou and the surrounding areas with severe infectious diseases. Patients were included if they met the criteria for severe tetanus according to Ablett’s classification (grade III or IV). Severe tetanus was defined by muscle rigidity, severe spasticity, significant respiratory involvement, or autonomic instability requiring intensive care. Patients with mild tetanus managed outside of the ICU or with insufficient clinical data were excluded. Mild tetanus was defined as Ablett grade I–II, and was managed without the need for airway protection or continuous intravenous deep sedation. There were no predefined exclusion criteria for ICU admission based on age or comorbidities. Admission decisions were primarily based on disease severity. During the study period, ICU capacity was sufficient, and no patients were excluded from ICU admission due to resource limitations.

### Data collection

The data of all patients were collected from the electronic medical records and included the following: demographic and clinical characteristics, treatment and supportive care, laboratory characteristics. All laboratory variables reported in this study represent baseline values obtained at ICU admission. Complications and clinical outcomes were also recorded. This study was approved by the Ethics Committee of The Fifth People’s Hospital of Suzhou (Approval No. HX-2025-001-02).

### Study design

This was a retrospective observational cohort study. We conducted a retrospective review of the records of all adult patients (≥ 18 years) with severe tetanus who were admitted to the ICU from January 2014 to December 2024. Approval for the study was obtained from the institutional ethics review board, and the requirement for informed consent was waived due to the retrospective nature of the study.

### Statistical analysis

Data were expressed as mean ± standard deviation for normally distributed continuous variables and as median (interquartile range) for non-normally distributed continuous variables. Categorical variables were summarized as frequencies and percentages. Statistical analyses were performed using SPSS version 26.0 (IBM Corp., Armonk, NY, USA). All variables were summarized using descriptive statistics, and no inferential analyses were conducted.

## Results

### Demographic characteristics

Among the 36 patients (Table 1), the median age was 60.5 years, and the proportion of males was 72.2%. Most of the patients (83.3%) resided in rural areas. With respect to occupation, 19 patients (52.8%) were farmers, 13 (36.1%) were manual workers, and 4 (11.1%) were retired. The most common comorbidities were hypertension (13 patients [36.1%]), diabetes (6 patients [16.7%]), and chronic lung disease (4 patients [11.1%]).

**Table 1:** Baseline demographic and clinical characteristics of patients with severe tetanus on ICU admission (*n* = 36)

Characteristics	Patients (*n* = 36)
**Age**,** years**,** median (IQR)**	60.50 (50.5–72.3)
**Male sex**,** n (%)**	26 (72.2)
**Rural residence**,** n (%)**	30 (83.3)
**Occupation**,** n (%)**	
Farmer	19 (52.8)
Worker	13 (36.1)
Retired	4 (11.1)
**Comorbidities**,** n (%)**	
Chronic lung disease	4 (11.1)
Hypertension	13 (36.1)
Diabetes	6 (16.7)
Cardiovascular disease	1 (2.8)
Active cancer	1 (2.8)
**Type of wound**,** n (%)**	
Metal injury	16 (44.4)
Wooden injury	5 (13.9)
Surgical operation	3 (8.3)
Other causes	12 (33.3)
**Pre-injury tetanus vaccination status**,** n (%)**	
Vaccinated	0 (0)
Unvaccinated	29 (80.6)
Unknown	7 (19.4)
**Tetanus vaccination after injury**,** n (%)**	1 (2.8)
**Tetanus antitoxin (TAT) after injury**,** n (%)**	4 (11.1)
**Tetanus immune globulin (TIG) after injury**,** n (%)**	1 (2.8)
**Incubation period**,** days**,** mean ± SD**	12.39 ± 9.47
**Onset of hospital**,** days**,** mean ± SD**	2.50 ± 1.81
**Ablett classification**,** n (%)**	
Severe	32 (88.9)
Very severe	4 (11.1)
**Age-adjusted Charlson Comorbidity Index (ACCI) on ICU admission**,** mean ± SD**	2.89 ± 1.75
**APACHE II score on ICU admission**,** median (IQR)**	6.0 (4.0–7.3)
**SOFA score on ICU admission**,** mean ± SD**	2.53 ± 1.36

Data are presented as n (%) or mean ± SD or median (IQR) as appropriate. Pre-injury vaccination status and post-injury prophylaxis in Table 1 refer to documented history before ICU admission. Abbreviations: ACCI, Age-adjusted Charlson Comorbidity Index; APACHE II, Acute Physiology and Chronic Health Evaluation II; SOFA, Sequential Organ Failure Assessment; TAT, tetanus antitoxin; TIG, tetanus immune globulin; IQR, interquartile range; SD, standard deviation

At admission, 44.4% of patients had wounds from metal objects, 13.9% by wooden objects, and 8.3% had a recent surgical history. Additionally, one-third of patients had wounds of other origins, including firecracker-related injuries, animal-related injuries, and injuries of unknown origin (Table 1). The detailed distribution of injury sites is presented in Supplementary Table S1. Pre-injury vaccination history was categorized as vaccinated, unvaccinated, or unknown. Among the 36 severe patients, 29 (80.6%) were unvaccinated, none were vaccinated, and 7 (19.4%) had unknown vaccination status. Only one patient (2.8%) received tetanus vaccination and tetanus immune globulin (TIG) after injury. Tetanus antitoxin (TAT) was given to four patients (11.1%). The mean length of incubation, which was defined as the time between the injury and the appearance of symptoms, was 12.39 ± 9.47 days. Patients were admitted after symptom onset, with a mean onset-to-admission interval of 2.50 ± 1.81 days.

**Fig. 1:**
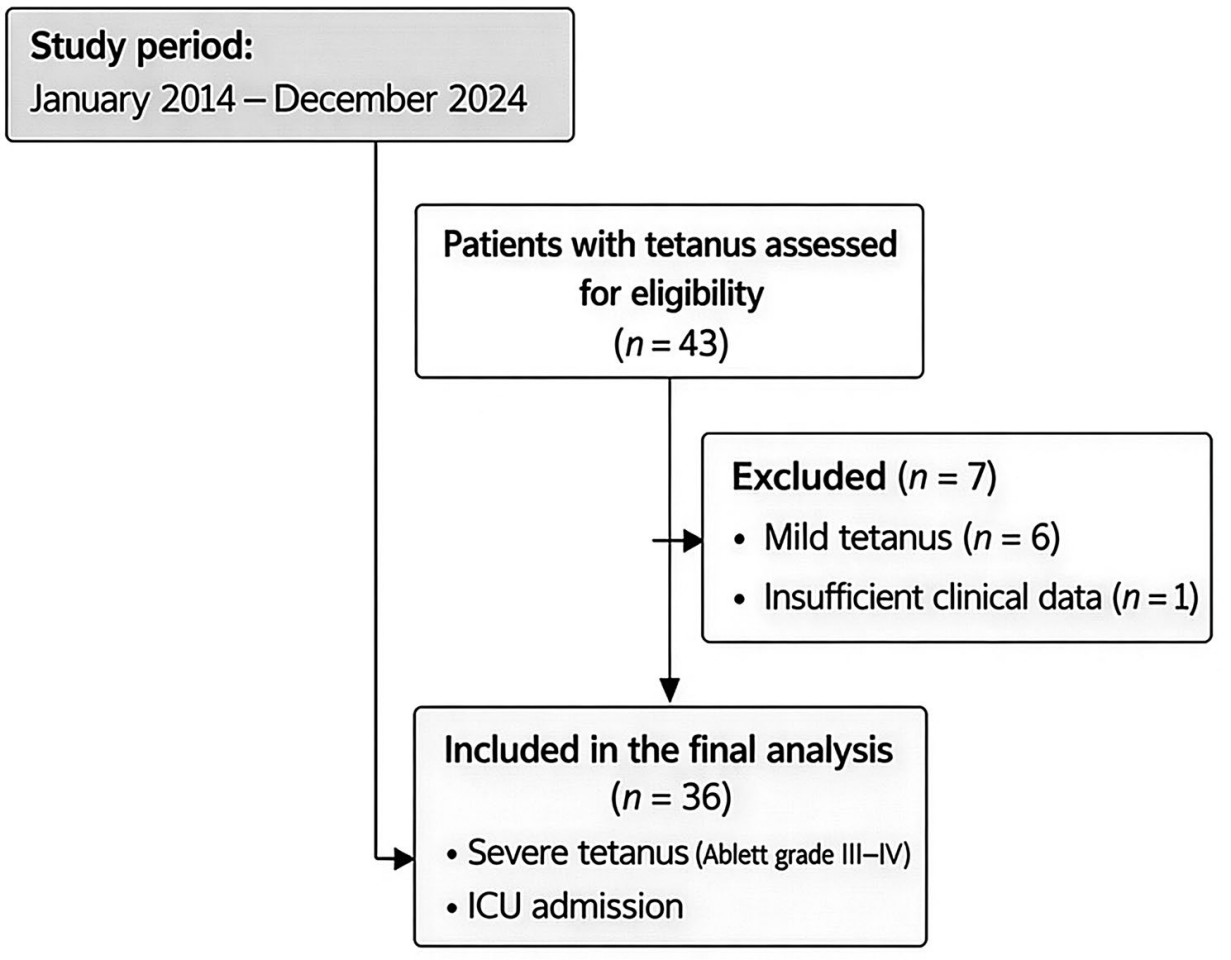
Flowchart of patient selection. A total of 43 patients with tetanus were assessed for eligibility between January 2014 and December 2024. Seven patients were excluded, including six patients with mild tetanus and one patient with insufficient clinical data. The final cohort consisted of 36 patients with severe tetanus (Ablett grade III-IV) admitted to the ICU

Between January 2014 and December 2024, 43 tetanus cases were identified hospital-wide. Six cases met the definition of mild tetanus and were managed outside the ICU, 1 case was excluded because of insufficient clinical data, and 36 severe cases (Ablett grade III–IV) were admitted to the ICU and included in this cohort (Fig. 1). Most of the cases were classified as Ablett grade III (32 patients, 89%), while 4 patients (11%) were classified as grade IV (Table 1). Upon admission, the disease severity scores were comparatively low, with a median APACHE II score of 6.0 (IQR, 4.0–7.3) and a mean SOFA score of 2.53 ± 1.36. Comorbidity burden was assessed using the age-adjusted Charlson Comorbidity Index, with a mean score of 2.89 ± 1.75.

### Clinical manifestations of tetanus

**Fig. 2:**
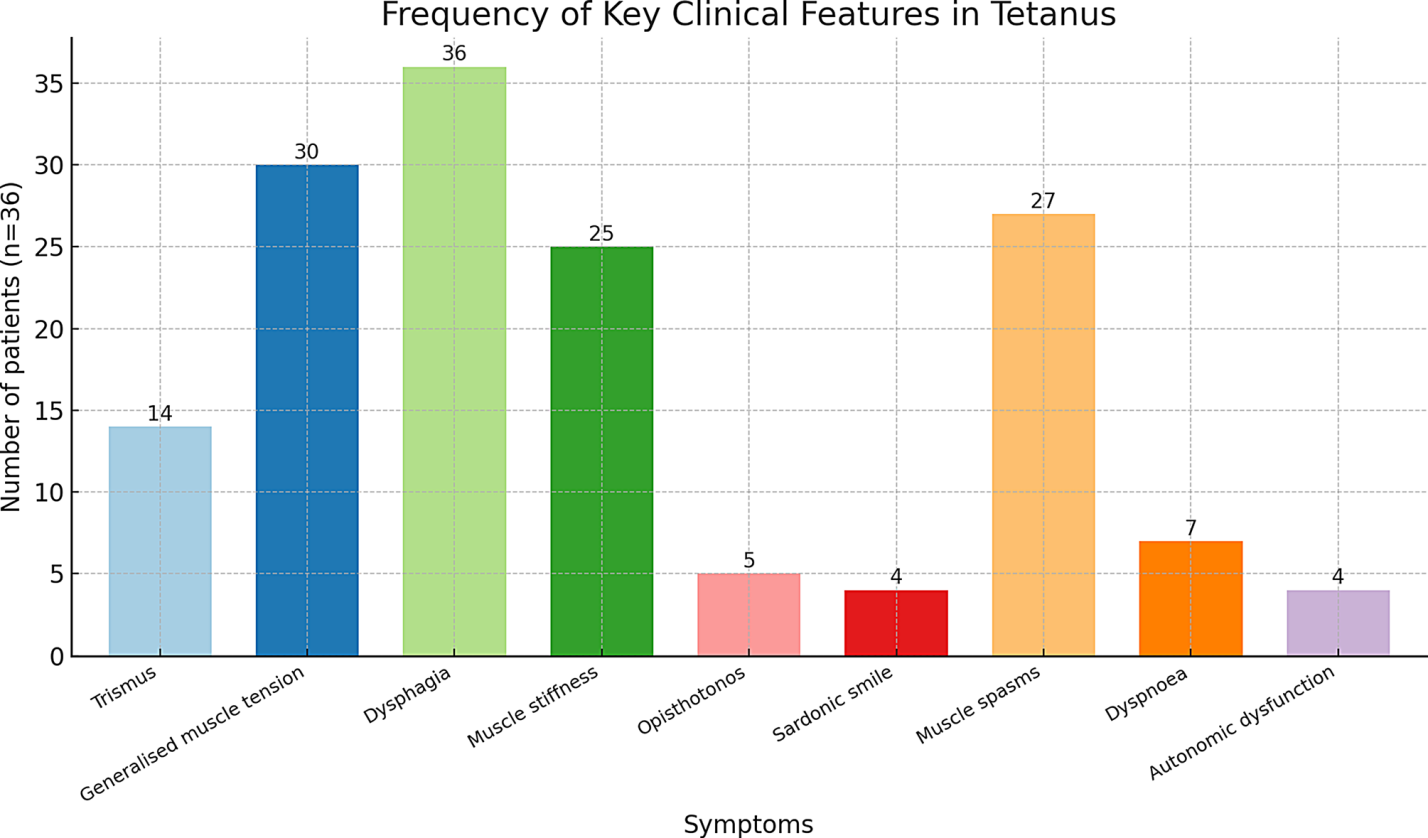
Frequency of key clinical manifestations in patients with severe tetanus (*n* = 36). This bar chart illustrates the number of patients presenting with major clinical features, including generalized muscle tension, dysphagia, muscle stiffness, opisthotonos, sardonic smile, muscle spasms, dyspnea, and autonomic dysfunction

As shown in Fig. 2, the most common symptom was dysphagia, which occurred in all 36 patients (100%). Generalized muscle tension was observed in 30 patients (83.3%). Additionally, muscle spasms were observed in 27 patients (75%) upon admission. Among these patients, 25 (69.4%) had marked muscle stiffness of the neck or limbs.

In this cohort, classical manifestations of tetanus were less frequently observed. Trismus at presentation was observed in 14 patients (38.9%) (Fig. 2). Dyspnoea was reported in 7 patients (19.4%), primarily due to laryngeal spasm or chest wall rigidity. Opisthotonos, characterized by abnormal extensor muscle spasms with severe backward arching, was present in 5 patients (13.9%). Four of the patients (11.1%) had sardonic smile. Similarly, 4 patients (11.1%) demonstrated clear signs of autonomic nervous system dysfunction, including arrhythmia, hyperthermia, or marked blood pressure instability before sedation.

### Treatment and supportive care

Regarding treatment, all patients received standard wound care and antimicrobial therapy with penicillin and metronidazole. Among the 36 patients, four received tetanus antitoxin (TAT) alone, 15 received tetanus immune globulin (TIG) alone, and 17 received both TAT and TIG (the use of TAT during the research period was due to the historical availability and resource constraints at that time). Furthermore, after admission, 10 patients (27.8%) required surgical debridement to remove necrotic tissue. Most patients required deep sedation and muscle relaxants to control severe spasticity, and the median duration of sedation was 20 days (IQR, 13.0–28.0 days). Thirty-one patients (86.1%) received benzodiazepines as part of the sedation regimen, with an median daily dose of 200 mg/day (IQR, 180.0–220.0 mg/day), and continuous remifentanil infusion was administered to 31 patients (86.1%) (Table 2). A total of 22 patients (61.1%) with persistent muscle rigidity and spasticity received vecuronium bromide. Additionally, eight patients (22.2%) required other agents, such as chlorpromazine and promethazine, for refractory agitation. Magnesium sulfate was not routinely used in this cohort due to institutional clinical practice and physician preference, together with concern about potential adverse effects such as hypotension and respiratory depression.

**Table 2:** Treatment and supportive care of patients with severe tetanus during ICU stay (*n* = 36)

Treatment	Total (*n* = 36)	Survivors (*n* = 30)	Non-survivors (*n* = 6)
**Antibiotic therapy, n (%)**			
Penicillin and Metronidazole	36 (100.0)	30 (100.0)	6 (100.0)
**Passive immunization strategy**,** n (%)**			
Tetanus antitoxin (TAT) alone	4 (11.1)	3 (10.0)	1 (16.7)
Tetanus immune globulin (TIG) alone	15 (41.7)	14 (46.7)	1 (16.7)
TAT + TIG	17 (47.2)	13 (43.3)	4 (66.7)
**Debridement after admission**,** n (%)**	10 (27.8)	10 (33.3)	0 (0.0)
**Sedation and muscle relaxation**,** n (%)**			
Benzodiazepines	31 (86.1)	26 (86.7)	5 (83.3)
Chlorpromazine and Promethazine	8 (22.2)	6 (20.0)	2 (33.3)
Vecuronium bromide	22 (61.1)	19 (63.3)	3 (50.0)
Remifentanil	31 (86.1)	28 (93.3)	3 (50.0)
**Respiratory support**,** n (%)**			
Non-invasive mechanical ventilation	6 (16.7)	3 (10.0)	3 (50.0)
Invasive mechanical ventilation	30 (83.3)	27 (90.0)	3 (50.0)
**Duration of continuous sedation**,** days**,** median (IQR)**	20 (13.0–28.0)	20.5 (14.0–30.0)	4 (3.0–7.0)
**Benzodiazepines dose**,** mg/day**,** median (IQR)**	200 (180.0–220.0)	220 (180.0–240.0)	130 (80.0–180.0)
**Duration of mechanical ventilation**,** days**,** median (IQR)**	21 (14.0–30.0)	22.5 (15.0–30.0)	4 (3.0–8.0)
**Tracheostomy**,** n (%)**	23 (63.9)	22 (73.3)	1 (16.7)
**Time to tracheostomy**,** days from ICU admission**,** median (IQR)**	7 (5.0–7.0)	7 (5.0–7.0)	3.5 (3.0–4.0)
**Beta-blockers**,** n (%)**	5 (13.9)	3 (10.0)	2 (33.3)
**Vasopressor medications**,** n (%)**	11 (30.6)	9 (30.0)	2 (33.3)
**Duration of vasopressor medication**,** days**,** mean ± SD**	2.19 ± 4.34	3.10 ± 4.96	1.00 ± 1.79
**Low-molecular-weight heparin**,** n (%)**	36 (100.0)	30 (100.0)	6 (100.0)

Data are presented as n (%) or mean ± SD or median (IQR) as appropriate. Passive immunization strategies shown in Table 2 refer to treatments administered during ICU stay. Abbreviations: TAT, tetanus antitoxin; TIG, tetanus immune globulin; IQR, interquartile range; SD, standard deviation

A large number of patients required invasive respiratory support due to respiratory failure caused by muscle stiffness. Thirty patients (83.3%) underwent endotracheal intubation (ETI) followed by invasive mechanical ventilation (IMV). Mechanical ventilation was prolonged, with a median duration of 21 days (IQR, 14.0–30.0 days) (Table 2). During the ICU stay, 23 patients (63.9%) required tracheostomy for long-term airway management, and the median time to tracheostomy was 7 days from ICU admission (IQR, 5.0–7.0 days). Six patients (16.7%) were initially managed with non-invasive ventilation (Table 2). Few patients required medication to control autonomic instability. Five patients (13.9%) received beta-blockers to reduce sympathetic overactivity (Table 2). Vasopressor support was given to 11 patients (30.6%) with hypotension or shock, with a mean duration of 2.19 ± 4.34 days. All patients received low-molecular-weight heparin for venous thromboembolism prophylaxis throughout their ICU stay (100%; Table 2).

### Laboratory findings

Key laboratory findings following ICU admission are detailed in Table 3 and are presented descriptively without inferential analysis. Upon ICU admission, patients had significant hematologic abnormalities, as well as inflammatory, organ-specific, cardiac, and respiratory abnormalities. Leukocytosis (47.2%; median 9.15 × 10⁹/L) with neutrophilia (55.6%; median 7.02 × 10⁹/L) and lymphopenia (36.1%; mean 1.40 ± 0.92 × 10⁹/L) was common. Anemia was frequent, affecting 52.8% of patients, with a median hemoglobin level of 125.5 g/L, while platelet counts were mostly preserved, with thrombocytopenia occurring in only 8.3% of patients. Coagulation abnormalities were observed, including prolonged prothrombin time in 61.1% of patients and elevated activated partial thromboplastin time in 33.3%. D-dimer levels were elevated in 52.8% of patients (mean 4.86 ± 7.17 µg/L). These findings indicate a systemic inflammatory response, characterized by elevated C-reactive protein (CRP) levels in 75% of patients.

**Table 3:** Laboratory characteristics of patients with severe tetanus on ICU admission (*n* = 36)

Characteristics	Total (*n* = 36)	Survivors (*n* = 30)	Non-survivors (*n* = 6)
**Blood routine**			
Leukocytes, ×10⁹/L, median (IQR)	9.15 (7.28–12.72)	9.66 (7.43–12.84)	7.08 (5.67–10.66)
Leukocytosis, n (%)	17 (47.2)	15 (50.0)	2 (33.3)
Neutrophils, ×10⁹/L, median (IQR)	7.02 (5.38–10.34)	7.09 (5.46–10.07)	5.49 (4.79–8.21)
Neutrophilia, n (%)	20 (55.6)	18 (60.0)	2 (33.3)
Lymphocytes, ×10⁹/L, mean ± SD	1.40 ± 0.92	1.47 ± 0.96	1.03 ± 0.63
Lymphopenia, n (%)	13 (36.1)	10 (33.3)	3 (50.0)
Platelets, ×10⁹/L, median (IQR)	203.00 (162.75–248.50)	203.50 (175.75–251.50)	175.50 (140.75–208.75)
Thrombocytopenia, n (%)	3 (8.3)	2 (6.7)	1 (16.7)
Hemoglobin, g/L, median (IQR)	128.50 (104.75–137.75)	129.50 (107.00–136.25)	109.00 (102.00–142.25)
Anemia, n (%)	19 (52.8)	15 (50.0)	4 (66.7)
**Coagulation function**			
APTT, s, median (IQR)	34.70 (30.00–39.83)	34.15 (29.80–39.48)	36.65 (34.78–39.58)
Prolonged APTT, n (%)	12 (33.3)	10 (33.3)	2 (33.3)
Shortened APTT, n (%)	2 (5.6)	2 (5.6)	0 (0.0)
Prothrombin time, s, median (IQR)	13.85 (12.73–14.43)	13.70 (12.57–14.40)	14.25 (13.95–14.47)
Prolonged prothrombin time, n (%)	22 (61.1)	17 (56.7)	5 (83.3)
D-dimer, µg/L, mean ± SD	4.86 ± 7.17	5.60 ± 7.65	1.13 ± 0.95
Elevated D-dimer, n (%)	19 (52.8)	17 (56.7)	2 (33.3)
**Liver function**			
Total bilirubin, µmol/L, mean ± SD	17.76 ± 30.37	12.77 ± 5.91	42.67 ± 73.21
Hyperbilirubinemia, n (%)	4 (11.1)	3 (10.0)	1 (16.7)
Albumin, g/L, median (IQR)	35.50 (30.40–38.85)	35.70 (30.62–38.95)	35.35 (30.37–36.88)
Hypoalbuminemia, n (%)	31 (86.1)	26 (86.7)	5 (83.3)
ALT, U/L, mean ± SD	52.06 ± 51.05	58.13 ± 53.73	21.67 ± 13.17
Elevated ALT, n (%)	13 (36.1)	13 (43.3)	0 (0.0)
AST, U/L, mean ± SD	56.22 ± 50.83	59.00 ± 50.53	42.33 ± 54.80
Elevated AST, n (%)	17 (47.2)	16 (53.3)	1 (16.7)
**Kidney function**			
Serum creatinine, µmol/L, mean ± SD	79.09 ± 37.69	80.89 ± 40.68	70.08 ± 15.11
Elevated creatinine, n (%)	3 (8.3)	3 (10.0)	0 (0.0)
Blood urea nitrogen, mmol/L, mean ± SD	7.79 ± 3.74	7.52 ± 3.96	9.13 ± 2.10
Elevated blood urea nitrogen, n (%)	9 (25.0)	8 (26.7)	1 (16.7)
**Cardiac injury**			
NT-proBNP, pg/mL, mean ± SD	673.83 ± 935.35	690.47 ± 992.81	590.67 ± 630.28
Elevated NT-proBNP, n (%)	25 (69.4)	21 (70.0)	4 (66.7)
LDH, U/L, mean ± SD	418.00 ± 231.98	412.43 ± 228.25	445.83 ± 270.95
Elevated LDH, n (%)	20 (55.6)	16 (53.3)	4 (66.7)
CK, U/L, mean ± SD	748.97 ± 1571.01	764.70 ± 1626.94	760.33 ± 1387.10
Elevated CK, n (%)	15 (41.7)	13 (43.3)	2 (33.3)
**Respiratory Function**			
PaCO₂, mmHg, median (IQR)	38.65 (36.73–46.35)	38.65 (35.60–45.75)	38.65 (37.33–46.65)
Increased PaCO₂, n (%)	10 (27.8)	8 (26.7)	2 (33.3)
Decreased PaCO₂, n (%)	7 (19.4)	7 (23.3)	0 (0.0)
PaO₂/FiO₂ ratio, mean ± SD	232.90 ± 80.43	235.30 ± 80.89	220.94 ± 84.39
Decreased PaO₂/FiO₂ ratio, n (%)	34 (94.4)	28 (93.3)	6 (100.0)
**Inflammatory biomarker**			
CRP, mg/L, mean ± SD	66.46 ± 68.58	75.56 ± 70.22	22.47 ± 39.58
Elevated CRP, n (%)	27 (75.0)	23 (76.7)	4 (66.7)

Data are presented as n (%) or mean ± SD or median (IQR) as appropriate. Abnormal values were defined according to local laboratory reference ranges. Abbreviations: APTT, activated partial thromboplastin time; ALT, alanine aminotransferase; AST, aspartate aminotransferase; CK, creatine kinase; CRP, C-reactive protein; LDH, lactate dehydrogenase; NT-proBNP, N-terminal pro–B-type natriuretic peptide; IQR, interquartile range; SD, standard deviation

Hypoalbuminemia was nearly universal (86.1%; median albumin, 35.5 g/L), with frequent mild elevations in hepatic enzymes (ALT, 36.1%; AST, 47.2%) and clinically significant hyperbilirubinemia was rare (11.1%). Renal impairment was not common at presentation, with elevated creatinine observed in 8.3% and mildly increased blood urea nitrogen levels in 25%. Levels of NT-proBNP were elevated in 69.4% (mean 673.83 ± 935.35 pg/mL) and lactate dehydrogenase levels were elevated in 55.6% of patients (mean 418.0 ± 232.0 U/L). Similarly, creatine kinase levels were elevated in 41.7% (mean 749.0 ± 1571.0 U/L). Impaired oxygenation was common, and reduced PaO₂/FiO₂ ratios were observed in 94.4% of patients (mean 232.9 ± 80.4). There were mixed ventilatory abnormalities, including hypercapnia in 27.8% and hypocapnia in 19.4% of patients, with a median PaCO₂ of 38.65 mmHg.

### Complications and outcomes

As shown in Table 4, thirty patients (83.3%) developed one or more infections during their ICU stay. Pulmonary infections were the most common complication, observed in 29 patients (80.6%). Thirteen patients (36.1%) developed wound-site infections despite appropriate wound treatment. However, only one case (2.8%) met the diagnostic criteria for ventilator-associated pneumonia, while other cases were classified as clinically diagnosed lower respiratory tract infections. It was observed that one case (2.8%) of bloodstream infection was present, alongside three cases (8.3%) of urinary tract infection. Importantly, more than half of the patients (55.6%) had cultures positive for drug-resistant organisms meeting local criteria for antimicrobial resistance. Most of the drug-resistant pathogens identified were multidrug-resistant Gram-negative bacteria, including *Acinetobacter baumannii*, *Klebsiella pneumoniae*, *Pseudomonas aeruginosa*,* Escherichia coli*,* and other Gram-negative bacteria* (detailed information on drug-resistant pathogens is provided in Supplementary Table S2). With such a high burden of infection, antibiotic therapy was prolonged, with a mean duration of 21.0 ± 10.7 days (Table 4).

**Table 4:** Complications and clinical outcomes of patients with severe tetanus during ICU stay (*n* = 36)

Complications and outcomes	Total (*n* = 36)	Survivors (*n* = 30)	Non-survivors (*n* = 6)
**Infections**,** n (%)**	30 (83.3)	25 (83.3)	5 (83.3)
Pulmonary infection	29 (80.6)	27 (90.0)	2 (33.3)
Wound infection	13 (36.1)	12 (40.0)	1 (16.7)
Bloodstream infection	1 (2.8)	1 (3.3)	0 (0.0)
Urinary tract infection	3 (8.3)	2 (6.7)	1 (16.7)
Ventilator-associated pneumonia	1 (2.8)	1 (3.3)	0 (0.0)
Drug-resistant bacterial infection	20 (55.6)	20 (66.7)	0 (0.0)
**Duration of antibiotic therapy**,** days**,** mean ± SD**	21.0 ± 10.7	24.2 ± 8.8	3.4 ± 1.1
**Post-exposure tetanus vaccination after injury**,** n (%)**			
Vaccinated	1 (2.8)	1 (3.3)	0 (0.0)
Unvaccinated	29 (80.6)	28 (93.3)	1 (16.7)
Unknown	6 (16.7)	1 (3.3)	5 (83.3)
**Laryngospasm / airway complications**,** n (%)**	7 (19.4)	6 (20.0)	1 (16.7)
**Autonomic instability**,** n (%)**	5 (13.9)	3 (10.0)	2 (33.3)
**Acute kidney injury**,** n (%)**	1 (2.8)	1 (3.3)	0 (0.0)
**Continuous renal replacement therapy (CRRT)**,** n (%)**	1 (2.8)	1 (3.3)	0 (0.0)
**Deep vein thrombosis**,** n (%)**	1 (2.8)	1 (3.3)	0 (0.0)
**Multiple organ failure**,** n (%)**	1 (2.8)	0 (0.0)	1 (16.7)
**ICU length of stay**,** days**,** median (IQR)**	27.5 (14.0–35.0)	30.5 (24.0–37.0)	4 (2.0–4.0)
**ICU mortality**,** n (%)**	6 (16.7)	0 (0.0)	6 (100.0)

Data are presented as n (%) or mean ± SD or median (IQR) as appropriate. Patients may have more than one complication. Abbreviations: CRRT, continuous renal replacement therapy; ICU, intensive care unit; IQR, interquartile range; SD, standard deviation

Clinically significant non-infectious complications were relatively uncommon. In our study, 7 patients (19.4%) developed laryngospasm accompanied by respiratory distress. Five patients (13.9%) developed severe autonomic dysfunction manifested by labile blood pressure and heart rate that needed treatment. One patient (2.8%) developed acute kidney injury during the ICU stay and required renal support with continuous renal replacement therapy. One patient (2.8%) developed a deep venous thrombosis despite prophylaxis, while another patient (2.8%) progressed to multiple organ failure.

The median length of ICU stay was 27.5 days (IQR, 14.0–35.0 days), indicating a prolonged ICU stay (Table 4). Despite aggressive critical care management, 6 patients died, resulting in an in-hospital mortality rate of 16.7%. In contrast, 30 patients (83.3%) survived to hospital discharge. When stratified by survival status, differences in the distribution of treatments and complications were observed between survivors and non-survivors, although no formal statistical comparisons were performed due to the limited sample size.

## Discussion

This study aimed to describe the clinical presentation and outcomes of severe tetanus. Our cohort was predominantly composed of older male patients from rural areas, many of whom had a history of occupational exposure and had not received adequate post-exposure prophylaxis. This is consistent with findings from previous reviews and studies [8, 9]. It is remarkable that only one individual was given the standard post-exposure tetanus vaccination and tetanus immunoglobulin (TIG). Most others had incomplete vaccinations histories or had never been vaccinated. These findings highlight that certain populations remain susceptible to tetanus. There is a need to improve vaccination strategies and post-exposure prophylaxis in elderly people working in high-risk occupations [10].

Vaccination records were incomplete in many patients. However, most severe cases were either unvaccinated or had unknown vaccination status, suggesting a possible association between inadequate immunization and disease severity. Despite the extremely low rate of post-exposure vaccination in our cohort, the observed mortality rate (16.7%) was comparable to that reported in other studies [11]. This may reflect the substantial impact of advances in intensive care management, including early airway protection, prolonged mechanical ventilation, and comprehensive supportive care. However, post-exposure prophylaxis primarily aims to neutralize circulating toxin and prevent disease progression, rather than reverse established neurotoxic effects. Therefore, its impact on mortality may be limited once severe tetanus has developed.

Clinically, dysphagia and generalized muscle tension were more commonly seen than the classical manifestations such as trismus and opisthotonos. Due to these unusual symptoms, diagnosis may be delayed, especially in areas where tetanus is rarely seen. Although the clinical classification is quite serious, the APACHE II score and SOFA scores at admission were fairly low, suggesting that early systemic organ failure is relatively uncommon at presentation. The distinct pathophysiology of tetanus is evidenced by its neuromuscular severity and lack of systemic organ dysfunction. High clinical suspicion should be maintained in patients with unexplained dysphagia and muscle tension [12].

In this study, all patients received antibiotic therapy consisting of a combination of penicillin and metronidazole, administered to reduce the bacterial load at the wound site. The mean duration of this therapy was 21.0 ± 10.7 days. This may reflect the increased antimicrobial resistance and higher burden of secondary infections associated with prolonged stays in intensive care units, rather than treatment directed solely at Clostridium tetani. In addition, almost all patients required deep sedation, with therapy being based on benzodiazepines and opioids. More than half of the patients received neuromuscular blocking agents because of drug resistant muscle rigidity. Over 80% of patients required invasive mechanical ventilation for a median duration of over 3 weeks. The high rate of tracheostomy likely reflects prolonged ventilator dependence and ongoing airway management needs. According to An et al. [13], our findings are consistent with previous reports. It also confirmed that the tetanus-induced respiratory failure has mainly neuromuscular origin and frequently is persistent in nature. Therefore, adequate planning for prolonged ventilatory support and improved airway management is warranted. On the other hand, autonomic instability remains a life-threatening complication of severe tetanus. In our cohort, only a minority of patients required beta-blockers, suggesting that adequate sedation, reduction of external stimuli, and ventilatory support controlled sympathetic overactivity in many cases.

Referring to the laboratory findings, many patients had leukocytosis, elevated CRP, hypoalbuminemia, and impaired oxygenation, indicating a systemic inflammatory response and metabolic stress associated with severe tetanus. In our cohort, the prevalence of hypoalbuminemia reached 86.1%. However, serum albumin is a non-specific marker influenced by inflammation, baseline nutritional status, comorbidity burden, and fluid balance. As systematic nutritional markers were not consistently available, we could not distinguish inflammation-driven hypoalbuminemia from malnutrition-related hypoalbuminemia. The elevation of NT-proBNP, CK, and LDH could be related to autonomic instability and sustained muscle activity. The frequency of infectious complications was significantly higher in our cohort, most notably pulmonary infections, with over half of patients carrying resistant strains. Prolonged usage of antibiotics along with increased stay in the ICU may contribute to this. The observed in-hospital mortality rate of 16.7% was comparable to that reported in earlier studies, suggesting that modern ICU management may have improved outcomes in severe tetanus [14–15]. Due to the descriptive nature of this study and the limited sample size, we did not perform survival-stratified analyses of laboratory parameters. Future studies are needed to determine their prognostic significance.

This study has several limitations. First, it was conducted in an ICU setting and included only adult patients (≥ 18 years), thereby excluding neonatal tetanus cases, which remain an important contributor to the global burden of disease in low-resource settings. Second, although all patients with severe tetanus (Ablett grade III–IV) were considered for ICU admission in our institution, the retrospective and single-center design introduces potential selection bias, and we cannot fully exclude the possibility that some patients were not admitted to the ICU due to clinical judgment, resource limitations, or underlying conditions. These factors may limit the generalizability of our findings. Moreover, missing documentation of pre-injury vaccination records in some patients limited the evaluation of the baseline immune status. This study also did not assess the long-term functional and neurological outcomes of survivors of severe tetanus after discharge, which limits the understanding of overall disease burden.

## Conclusion

Taken together, tetanus can be a fatal condition. However, in well-resourced settings with good access to intensive care, early airway protection, wound debridement, toxin neutralisation, individualised sedation, and close infection surveillance and management may lead to favourable outcomes. Patients with trismus, dysphagia, muscle stiffness or spasms should be viewed with a high index of suspicion by clinicians. It is particularly important to strengthen pre-exposure vaccination and post-exposure prophylaxis strategies against tetanus.

## Supplementary Information

Below is the link to the electronic supplementary material.

Supplementary Material 1

Supplementary Material 2

## Data Availability

The datasets used and/or analyzed during the current study are not publicly available due to patient privacy and ethical restrictions. De-identified data may be available from the corresponding author upon reasonable request and subject to institutional ethics approval.

## Abbreviations

ACCI: Age-adjusted Charlson Comorbidity Index
AKI: Acute kidney injury
ALT: Alanine aminotransferase
APACHE II: Acute Physiology and Chronic Health Evaluation II
AST: Aspartate aminotransferase
CK: Creatine kinase
CRP: C-reactive protein
DVT: Deep venous thrombosis
ETI: Endotracheal intubation
FiO₂: Fraction of inspired oxygen
ICU: Intensive care unit
IMV: Invasive mechanical ventilation
IQR: Interquartile range
LDH: Lactate dehydrogenase
LMWH: Low-molecular-weight heparin
NT-proBNP: N-terminal pro–B-type natriuretic peptide
PaCO₂: Partial pressure of arterial carbon dioxide
PaO₂: Partial pressure of arterial oxygen
SD: Standard deviation
SOFA: Sequential Organ Failure Assessment
SPSS: Statistical Package for the Social Sciences
TAT: Tetanus antitoxin
TIG: Tetanus immune globulin
VAP: Ventilator-associated pneumonia
