# Supplementary material for: Clinical characteristics, management, and outcomes of severe tetanus in the intensive care unit
Source: BMC Infect Dis. 2026 May 28;26:1403. doi: 10.1186/s12879-026-13697-6 (PMC13411524; doi:10.1186/s12879-026-13697-6)
Supplement: Supplementary file 1 — Supplementary Material 1 [file 12879_2026_13697_MOESM1_ESM.docx]

**Supplementary Table S1. Distribution of injury sites in patients with severe tetanus (n = 36)**

| **Site of injury** | **No. of patients** | **Percentage (%)** |
| --- | --- | --- |
| Lower extremity | 13 | 36.1 |
| Upper extremity | 16 | 44.4 |
| Head/face | 2 | 5.6 |
| Trunk | 4 | 11.1 |
| Multiple | 5 | 13.9 |
| Unknown | 3 | 8.3 |

*Patients with multiple injury sites were counted in more than one category.*
