# Supplementary material for: Clinical characteristics, management, and outcomes of severe tetanus in the intensive care unit
Source: BMC Infect Dis. 2026 May 28;26:1403. doi: 10.1186/s12879-026-13697-6 (PMC13411524; doi:10.1186/s12879-026-13697-6)
Supplement: Supplementary file 2 — Supplementary Material 2 [file 12879_2026_13697_MOESM2_ESM.docx]

**Supplementary Table S2. Distribution of drug-resistant pathogens in patients with severe tetanus (n = 36)**

| **Pathogen** | **Number of isolates** | **Resistance profile** |
| --- | --- | --- |
| **Acinetobacter baumannii, n (%)** | 9 (25.0) | MDR |
| **Klebsiella pneumoniae, n (%)** | 8 (22.2) | ESBL |
| **Pseudomonas aeruginosa, n (%)** | 9 (25.0) | MDR |
| **Escherichia coli, n (%)** | 6 (16.7) | ESBL |
| **Other Gram-negative bacteria, n (%)** | 5 (13.9) | MDR |
| **Staphylococcus aureus, n (%)** | 6 (16.7) | MRSA |

Abbreviations: MDR, multidrug-resistant; ESBL, extended-spectrum β-lactamase; MRSA, methicillin-resistant Staphylococcus aureus.
